# Completing the Puzzle: A Cluster of Hunting Dogs with Tick-Borne Illness from a Fishing Community in Tobago, West Indies

**DOI:** 10.3390/pathogens13020161

**Published:** 2024-02-10

**Authors:** Roxanne A. Charles, Patricia Pow-Brown, Annika Gordon-Dillon, Lemar Blake, Soren Nicholls, Arianne Brown-Jordan, Joanne Caruth, Candice Sant, Indira Pargass, Asoke Basu, Emmanuel Albina, Christopher Oura, Karla Georges

**Affiliations:** 1 School of Veterinary Medicine, Faculty of Medical Sciences, The University of the West Indies, St. Augustine, Trinidad and Tobago; patpowbrown@hotmail.com (P.P.-B.); lemar_blake@hotmail.com (L.B.); candice.sant@sta.uwi.edu (C.S.); indira.pargass@sta.uwi.edu (I.P.); asokebasu@gmail.com (A.B.); christopher.oura@sta.uwi.edu (C.O.); karla.georges@sta.uwi.edu (K.G.); 2 Animal Health Unit, Division of Food Security, Natural Resources, The Environment and Sustainable Development, Tobago House of Assembly, Milshirv Administrative Complex, Corner Milford & Shirvan Road, Tobago, Trinidad and Tobago; jambagovet@gmail.com (A.G.-D.); jocaruth@hotmail.com (J.C.); 3 Department of Preclinical Sciences, Faculty of Medical Sciences, The University of the West Indies, St. Augustine, Trinidad and Tobago; soren.nicholls@gmail.com (S.N.); arianne.brown-jordan@sta.uwi.edu (A.B.-J.); 4 Centre de coopération internationale en recherche agronomique pour le développement (CIRAD), DGDRS, 34000 Montpellier, France; emmanuel.albina@cirad.fr

**Keywords:** *Anaplasma*, *Amblyomma*, *Babesia*, *Ehrlichia*, *Hepatozoon*, *Rhipicephalus*, ticks, tick-borne diseases, Tobago, West Indies

## Abstract

Eight hunting dogs were visited by a state veterinarian on the island of Tobago, Trinidad and Tobago, West Indies, as owners reported anorexia and paralysis in five of their dogs. The veterinarian observed a combination of clinical signs consistent with tick-borne illness, including fever, anorexia, anaemia, lethargy and paralysis. Blood and ticks were collected from each dog and submitted to a diagnostic laboratory for analysis. Microscopic analysis revealed a mixed infection of intracytoplasmic organisms consistent with *Babesia* spp. (erythrocyte) and *Ehrlichia* spp. (monocyte), respectively, from one dog, while a complete blood count indicated a regenerative anaemia (n = 1; 12.5%), non-regenerative anaemia (n = 4; 50%), neutrophilia (n = 3; 37.5%), lymphocytosis (n = 2; 25%), thrombocytopaenia (n = 3; 37.5%) and pancytopaenia (n = 1; 12.5%). DNA isolated from the eight blood samples and 20 ticks (16 *Rhipicephalus sanguineus* and 4 *Amblyomma ovale*) were subjected to conventional PCR and next-generation sequencing of the 16S rRNA and 18S rRNA gene for *Anaplasma/Ehrlichia* and *Babesia/Theileria/Hepatozoon*, respectively. The DNA of *Ehrlichia* spp., closely related to *Ehrlichia canis*, was detected in the blood of three dogs (37.5%), *Anaplasma* spp., closely related to *Anaplasma marginale*, in two (25%), *Babesia vogeli* in one dog (12.5%) and seven ticks (35%) and *Hepatozoon canis* and *Anaplasma* spp., in one tick (5%), respectively. These findings highlight the need to test both the vector and host for the presence of tick-borne pathogens when undertaking diagnostic investigations. Further studies are also warranted to elucidate the susceptibility of canids to *Anaplasma marginale.*

## 1. Background

Ticks are vectors of pathogenic viruses, bacteria and protozoa that affect animals and humans. The resulting diseases can severely impact the health and well-being of their affected hosts. The tick-borne pathogens (TBPs) of dogs are diverse, and the clinical signs may range from inapparent to acute illness. Considerable morbidity may include blood coagulopathies, anaemia, organ damage, leukopaenia, paresis, neurological deficits and mortality in untreated or complicated clinical cases [1,2,3,4].

The brown dog tick, *Rhipicephalus sanguineus*, is one of the main arthropod vectors responsible for the transmission of TBPs in dogs worldwide [5]. A previous study found that this tick is the most prevalent species found on dogs on the island of Tobago [6]. *Rhipicephalus sanguineus* ticks have been implicated in the transmission of a range of canine TBPs, including *Anaplasma platys, Babesia canis*, *Ehrlichia canis*, *Hepatozoon canis* and *Rickettsia* spp. [5,7]. This tick is also the vector of *B. canis, E. canis, *Ehrlichia* chaffeensis, *Ehrlichia* ewingii, Coxiella burnetti* and several spotted fever group (SFG) *Rickettsia* spp. in humans, notably *Rickettsia rickettsii, R. conorii* and *Rickettsia massiliae* [8,9,10,11].

*Amblyomma ovale* is also known to infest dogs on the island [6]. Immature stages feed mainly on birds and small rodents, while adults complete their life cycle on larger vertebrates, including canids [12]. Human infestations have been reported in the Americas, including Costa Rica, Panama and Brazil [13,14,15,16,17]. Infections of *A. ovale* with *H. canis* and the SFG agent, *R. parkeri* strain Atlantic rainforest, pathogens of canids and humans, respectively, have also been documented [17,18,19,20,21].

Canine babesiosis, caused by the protozoan parasites of the *Babesia* spp. (*B. gibsoni, B. canis, B. vogeli* and *B. rossi*), is an important tick-borne disease (TBD) of dogs, with several publications from the Caribbean [22]. *Babesia vogeli* is prevalent in the Caribbean and is often associated with a milder, sometimes subclinical infection when compared to the most virulent, *B. rossi*. Another protozoan parasite, *H. canis,* causes milder cases of anaemia and lethargy compared to its more virulent counterpart *Hepatozoon americanum* [23]. Unlike other TBPs, which are transmitted via the bite of infected ticks, *H. canis* is transmitted by the ingestion of infected ticks.

Canine monocytic ehrlichiosis (CME) is caused by the rickettsial organism *E. canis*. This Gram-negative, pleomorphic bacterium is found as membrane-bound morulae in the mononuclear cells of the infected host. Canine ehrlichiosis is a multi-systemic disease that can manifest as acute, subclinical or chronic forms. Acute disease is characterised by pyrexia, depression, anorexia, lethargy, lymphadenopathy, splenomegaly and haemorrhagic tendencies in the form of dermal ecchymoses, uveitis and epistaxis [24,25,26,27]. Similar signs are manifested in the chronic phase but may be more severe. Another obligate, intracellular rickettsial organism, *Anaplasma platys,* resides in the platelets of dogs [25,28]. It is vectored by *R. sanguineus* and causes canine cyclic thrombocytopaenia [24,29]. Co-infection with *E. canis* is frequently detected in domestic dogs, causing more severe signs, including thrombocytopaenia [30,31,32].

Tick infestations and TBDs of the canine population have been reported throughout the Caribbean [6,22,33,34,35,36,37]. However, very little or no reported data is available for some islands, including Tobago, the smaller of the two islands that comprise the Republic of Trinidad and Tobago. The islands are the southernmost in the Caribbean, with Tobago located 35 km northeast of Trinidad and to the southeast of Grenada. Tobago’s population is approximately 61,000 inhabitants within a land area of 300 km^2^. The climate is tropical with two seasons—wet (June to December) and dry (January to May). The canine population consists of both domesticated and stray dogs. Frequently, dogs are free to roam throughout the villages, interacting with other dogs and other species, including wildlife.

In November 2020, a cluster of dogs exhibiting clinical signs consistent with tick-borne illness was reported in Charlotteville, a small fishing village in Tobago. Dogs on this island are known to be infested with *R. sanguineus* and *A. ovale* ticks [6]. While diagnostic methods such as clinical presentation, microscopic detection of pathogens in peripheral blood smears, serological testing (lateral flow test kits) and response to chemotherapeutic agents are used to diagnose tick-borne illness in animals in Tobago, their sensitivity and specificity are not reliable. The aim of this study was therefore to use more sensitive molecular techniques, together with classical techniques, to detect and characterise the TBPs in a cluster of dogs suspected of having tick-borne illness and their ticks from a small village in Tobago.

## 2. Methods

### 2.1. Study Period and Location

**Figure 1:**
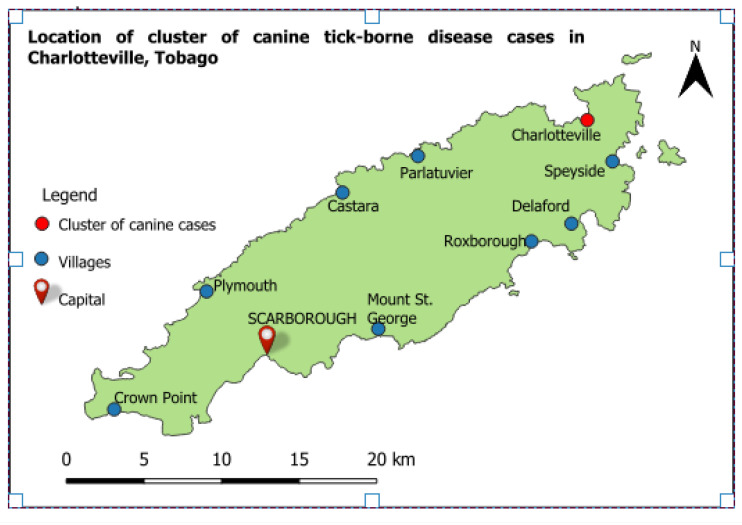
Map of Tobago showing the location of the cluster of suspected canine TBD cases in Charlotteville. Map generated using the free and open source QGIS software.

Samples were collected from dogs residing in Charlotteville, Tobago (11°19′32.0″N 60°32′49.7″W) in November 2020 (Figure 1).

### 2.2. Field Collection and Processing of Blood and Ticks

The veterinary officer for the area received a report that five dogs in the study area were exhibiting neurological signs related to paresis and recumbency. Further investigations were conducted to determine if these five dogs, three other dogs in close proximity and ticks infesting the dogs were positive for TBPs, as tick-borne illness was a primary differential. Blood and ticks (if present) were collected from all dogs in the cluster. A questionnaire with demographic data, signalment (age, sex and breed) and history of tick infestation and TBD was completed for each dog.

Blood from each dog was collected via cutaneous venepuncture of the cephalic vein and placed into an EDTA tube (purple top tube) and tube without anticoagulant (red top tube) for serum. Ticks attached to infested dogs were collected and placed in plastic vials with the tops punctured to allow entry of air. On the field, all blood and ticks were placed in a cooler with an icepack, followed by refrigeration at 4 °C within 2 h of collection. All samples were then transported within 14 h of collection to the University of the West Indies, School of Veterinary Medicine (UWI-SVM) in Trinidad for further analysis. Blood samples were analysed within 1 h of receipt from Tobago, while ticks were stored at −20 °C until further processing.

### 2.3. Diagnostic Testing

#### 2.3.1. Microscopic Examination of Blood

Thin blood smears were prepared and stained with Wright–Giemsa stain. Slides were examined using light microscopy under oil immersion (100× objective) using the Olympus BX41 microscope (Olympus Corporation, Tokyo, Japan). A sample was considered positive if any inclusion bodies morphologically consistent with trophozoites, merozoites, gamonts or bacteria were detected in any erythrocytes, leukocytes or platelets respectively.

#### 2.3.2. Microscopic Examination of Ticks

All ticks were morphologically identified to the species level using established taxonomic identification keys [38] and separated according to sex visually under a Olympus SZ2-ILST dissection microscope (Olympus Corporation, Tokyo, Japan) at a magnification of 56×.

#### 2.3.3. Complete Blood Count and Serum Biochemistry

Complete blood counts (CBCs) were performed using the IDEXX Procyte Haematology Analyser (IDEXX Laboratories, Incorporated, Maine, ME, USA) and serum biochemistry using the Mindray B-200 automated biochemistry analyser (Mindray Bio-Medical Electronics Co. Ltd., Shenzhen, China).

### 2.4. DNA Extraction and Quantification

For each dog, DNA was extracted from 100µL of EDTA blood using the Qiagen DNeasy Blood and Tissue Kit (Qiagen, Maryland, MD, USA) according to manufacturer’s instructions. The DNA was eluted in 200 µL elution buffer and stored at −20 °C until further analysis.

Before DNA extraction, individual ticks were washed in 70% ethanol, followed by 5% sodium hypochlorite, then rinsed with distilled water followed by a phosphate buffered saline (PBS) rinse for one minute. The ticks were then dried on sterile Whatman^®^ filter paper and placed into individually labelled sterile 2 mL Eppendorf tubes. Each tick was dissected into four parts with a sterile scalpel blade inside 2 mL Eppendorf tubes, using a new blade for each tick. A total of 180 µL of lysis buffer and 20 µL proteinase K (Qiagen, Maryland, MD, USA) was added to each tube then incubated at 56 °C overnight. Total DNA was then extracted using the Qiagen DNeasy Blood and tissue kit (Qiagen, Maryland, MD, USA), adjusted to 200 µL of buffer AE and stored at −20 °C until further use.

After extraction, DNA concentrations from the dog blood and ticks were determined by spectrophotometry (NanoDrop^®^ One C 2000 Spectrophotometer, Thermo Fisher Scientific, Madison, WI, USA). To minimise risk of contamination, DNA extractions, PCR preparation, PCR amplification and agarose gel electrophoresis were performed in separate rooms.

### 2.5. PCR Amplification of 16S rRNA and 18S rRNA

Individual PCR reactions of 25 µL consisted of 5 µL of extracted DNA, 12.5 µL of Chai 2× Master Mix (Chai Biotechnologies Inc., Santa Clara, CA, USA), 1 µM of each primer and 5.5 µL of PCR grade water (Sigma-Aldrich Inc. St. Louis, Missouri, MO, USA). For the detection of *Babesia* and *Hepatozoon* spp., primers RLB F2 and RLB R2 (Table 1) were used to amplify the 18S rRNA gene spanning the V4 hypervariable region [39]. The hypervariable V1 region of the 16S rRNA gene was amplified to detect *Anaplasma* and *Ehrlichia* DNA using primers B-GA1B and 16 S8FE [40] (Table 1). PCRs were performed using a Techne Flexigene Thermal Cycler (Techne, Cambridge, UK) with the following cycling parameters for both sets of primers: initial denaturation at 94 °C for 10 min, followed by 35 additional denaturation cycles at 94 °C for 30 s, annealing at 53 °C for 30 s and extension at 72 °C for 45 s. A final extension step was performed at 72 °C for 5 min. Samples were then held at 4 °C.

All PCR products were separated by electrophoresis through a 2% agarose gel in 1% TBE buffer impregnated with GelRed^®^ nucleic acid gel stain (Biotium Incorporated, Fremont, CA, USA) and visualised by UV illumination. The negative control was PCR grade water, and the positive controls were DNA isolated from the blood of confirmed *Babesia, Hepatozoon* and *Ehrlichia* positive animals.

### 2.6. Sequence Analysis of TBPs

To confirm the results obtained by PCR, 16S and 18S rRNA amplicons from canine blood and ticks were sequenced using a next-generation sequencing (NGS) approach utilising the Oxford Nanopore Technologies’ (ONT) GridION^®^ (Oxford, UK) at the Department of Pre-Clinical Sciences, Faculty of Medical Sciences, the University of the West Indies, Trinidad and Tobago, West Indies. The library was prepared using a ligation kit (SQK- LSK109, Oxford Nanopore Technology, Oxford, UK) and analysed with a FLOMIN106 flow cell (v9.4.1). Basecalling of the resultant Fast5 files was performed using Guppy (v6.3.8) to produce fastq files [16]. Quality checking was assessed using PycoQC (https://github.com/a-slide/pycoQC, accessed on 17 July 2023) and reference-based assembly of the files was performed with minimap2 (https://github.com/lh3/minimap2, accessed on 17 July 2023), samtools (https://github.com/samtools/, accessed on 17 July 2023) and bcftools (https://github.com/samtools/bcftools, accessed on 17 July 2023). Polishing was done using Medaka (https://github.com/nanoporetech/medaka, accessed on 17 July 2023). Resultant nucleotide sequences were compared with existing sequences in the GenBank database using the Basic Local Alignment Search Tool (BLAST) algorithm [42] on NCBI (https://blast.ncbi.nlm.nih.gov/Blast.cgi, accessed on 17 July 2023). Reference sequences from GenBank with a query coverage of 97–100% were compared to sequenced microorganisms for phylogenetic analysis.

### 2.7. Phylogenetic Analysis of TBPs

Partial 16S and 18S rRNA gene sequences derived in this study were aligned with reference sequences from GenBank with query coverage ranging from 97–100% identity with *B. vogeli* 18S rRNA and *A. marginale* and *E. canis* 16S rRNA using the Muscle algorithm. Molecular phylogenies were inferred from the two resulting data sets [(i) 49 partial 16S rRNA sequences from this study and GenBank, including *A. marginale, A. platys, A. phagocytoplilum, E. canis* and *Neorickettsia risticii* (Accession number: NR029162.1) as an outgroup, and (ii) 21 partial *B. vogeli* 18S rRNA sequences, including *Toxoplasma gondii* isolate, Tg10 (Accession number: KX008033.1) as the outgroup, using the MEGA11 software. The maximum-likelihood algorithm was selected based on best fit [43]. Data sets were sampled 1000 times for bootstrap value generation using the best fit substitution models, i.e., Tamura-Nei model for the 18S rRNA sequences and Kimura 2-parameter for the 16S rRNA sequences [44]. All sequences from this study were deposited in GenBank with the following accession numbers: OR077268-OR077273 and OR666420 for the protozoan TBPs and OR296878-OR296884 for the rickettsial pathogens.

## 3. Results

### 3.1. Clinical Signs, Tick Infestations and Haematological Findings

A cluster of eight dogs (six female and two male) ranging in age from 5 months to 8 years were sampled in this study. A summary of the clinical presentation of these dogs is provided in Table 2. The most prevalent clinical signs were visible weight loss (n = 6), paralysis (n = 5), anorexia (n = 5), anaemia (n = 6) and listlessness (n = 4). Two dogs exhibited dermal ecchymoses (Dogs 1 and 4), of which one of these dogs (Dog 4) also presented with uveitis. Another dog experienced stillbirths of full-term puppies (Dog 6). The temperatures of the dogs were not recorded at the time of sampling. Ectoparasites (20 ixodid ticks) were collected from six dogs (75%) and identified as adult *R. sanguineus* (n = 16; 11 male, five female) and *A. ovale* (n = 4; all female). Ticks were not observed on two (25%) dogs.

**Figure 2:**
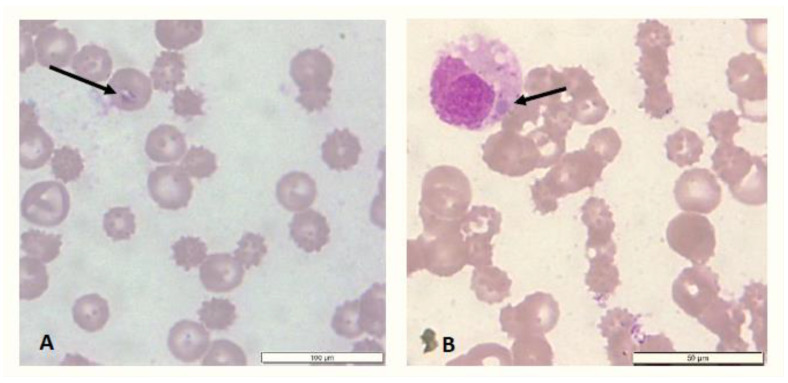
Giemsa-stained peripheral blood smear of a dog (Dog 5) from Charlotteville, Tobago, showing an intra-erythrocytic inclusion body (**A**) consistent with *Babesia vogeli* and an intracytoplasmic mononuclear inclusion body (**B**) consistent with *Ehrlichia canis* (indicated by arrows).

Microscopic examination of blood smears from one dog (Dog 5) revealed a mixed infection of rare intra-erythrocytic inclusion bodies consistent with *Babesia* spp. and a morula, consistent with *E. canis,* in the cytoplasm of a mononuclear cell (Figure 2). It should be noted that the blood of this dog was observed to be very ‘thin’ on venipuncture. The changes in the erythrogram of sampled dogs included anaemia, ranging from poorly regenerative (n = 1) to non-regenerative anaemia (n = 4) (Table 2). Changes in the leukogram included neutrophilia (n = 3), neutropaenia (n = 1), lymphocytosis (n = 2), lymphopaenia (n = 1), monocytosis (n = 2) thrombocytopaenia (n = 3) and pancytopaenia (n = 1). Blood references were based on Comazzi and Weiss [42,45]. The clinical outcomes of the eight cases are presented in Table 3. The youngest dog (Dog 1) and the oldest (Dog 8) died within a day and six weeks, respectively, of sampling. Although the clinical signs exhibited by these two dogs mirrored canine TBD cases, no TBPs were detected in their blood. Further, the cause of death in both cases was inconclusive since necropsies were not performed.

### 3.2. Molecular Detection of TBPs in Dog Blood

The overall frequency of TBPs in dog blood was 62.5% (five out of eight dogs) of those sampled. Single infections of *Ehrlichia* spp. (n = 3, 37.5%) and *Anaplasma* spp. (n = 2, 25%) were amplified in the blood of five dogs. A mixed infection of *B. vogeli* and *Ehrlichia* spp. was detected in one dog (12.5%) only. Moreover, the blood smear of this dog (Dog 5) was positive for *Babesia* and *Ehrlichia* spp.

Sequences of the *Anaplasma/Ehrlichia* 16S rRNA gene amplified in this study were 98–100% homologous to *E. canis* and 99–100% to *Anaplasma marginale* when compared to reference sequences previously deposited in GenBank. The *Babesia* spp. detected in the blood of Dog 5 showed a 97% similarity to *B. vogeli* sequences in GenBank. All pathogen sequences derived were deposited in GenBank under the following accession numbers: *Anaplasma* spp. (OR296882-OR296884), *B. vogeli* (OR666420) and *Ehrlichia* spp. (OR296878-OR296881).

### 3.3. TBPs in Ticks Infesting Dogs

Of the 20 ixodid ticks screened, TBPs were detected by PCR in eight (40%) individual ticks, from five (83.3%) of the six infested dogs. *Babesia vogeli* was detected in both *R. sanguineus* (n = 5, four male and one female) and *A. ovale* (n = 2, both female), while *H. canis* was amplified in only one *R. sanguineus* female tick. One dog (Dog 3) was host to six *R. sanguineus* ticks, of which *B. vogeli* DNA was amplified in two ticks and *H. canis* in one tick (Table 4). In another dog (Dog 7), two tick species consisting of four *R. sanguineus* and two *A. ovale* were detected. Of these six ticks, *B. vogeli* DNA was amplified in *R. sanguineus* (n = 2) and *A. ovale* (n = 1). Additionally, *A. marginale* DNA was also amplified in the same *A. ovale* tick from Dog 7 and *E. canis* DNA was amplified in the *A. ovale* ticks from Dog 8. A *Francisella*-like endosymbiont was detected in one *A. ovale* tick from Dog 6.

Sequences of the amplified *Babesia/Theileria/Hepatozoon* 18S rRNA gene were 98–99% identical to reference sequences in GenBank. The derived TBP sequences from ticks were assigned the following accession numbers: *Anaplasma* spp. (OR296884), *B. vogeli* (OR077267-OR077273) and *H. canis* (OR077267).

### 3.4. Comparison between the Presence of TBP DNA in Blood and Ticks from the Same Dog

Tick-borne pathogen DNA was amplified using PCR from the blood of five dogs (62.5%) and eight ticks (40%). However, different pathogens were detected in the blood versus ticks from some dogs. For example, *E. canis* was amplified only in the blood of Dog 6, while a *Francisella* spp. endosymbiont was detected in the sole tick found on this dog (data not included). In contrast, TBPs were not amplified in the blood of Dogs 1 and 7 but *B. vogeli* and *Anaplasma* spp. were amplified in some of the ticks infesting them. Tick-borne pathogens were amplified in both blood (*Anaplasma* spp.) and ticks (*B. vogeli*, *H. canis* and *Ehrlichia* spp.) of Dogs 3 and 8, respectively (Table 4).

### 3.5. Comparison among the Presence of TBP DNA in the Blood, Clinical Signs and Haematological Findings Presented in Each Dog

DNA homologous with *E. canis* (n = 3), *A. marginale* (n = 2) and *B. vogeli* (n = 1) was amplified from blood of five of the eight dogs in the Tobago cluster. Of the three *Ehrlichia* spp. positive blood samples (Dogs 4, 5 and 6), clinical signs included anaemia, anorexia, ecchymotic haemorrhages, listlessness and, in one case, uveitis, abortion and paralysis (Table 2). One of the *Ehrlichia* spp.-infected dogs was co-infected with *B. vogeli* (Dog 5). A similar clinical picture was presented in the two dogs with *Anaplasma* spp. (Dogs 3 and 8); however, dogs in which no TBP DNA was amplified (Dogs 1, 2 and 7) showed at least one clinical sign suggestive of TBD. The haematological data highlights non-regenerative to poorly regenerative anaemia, thrombocytopaenia and hyperproteinaemia being the most prevalent findings in the cluster of cases.

### 3.6. Sequence Analysis

Basic Local Alignment Search Tool (BLAST) analysis of the 16S rRNA isolates of *Ehrlichia* spp. from this study shared a 98–100% homology with isolates from India (KX364265.1), Thailand (AB287435.1) and Turkey (KY247110.1), while *Anaplasma* spp. shared a 99–100% homology with sequences from Puerto Rico (MK737024.1), Croatia (MN187218.1) and Iran (MK310488.1). For *B. vogeli*, positive amplicons shared a 97–98% sequence identity with sequences from China (HM590440.1), Egypt (AY371197.1) and Japan (AB083374.1). The best GenBank matches for the sequences from this study are shown in Table 5.

### 3.7. Phylogenetic Analysis

**Figure 3:**
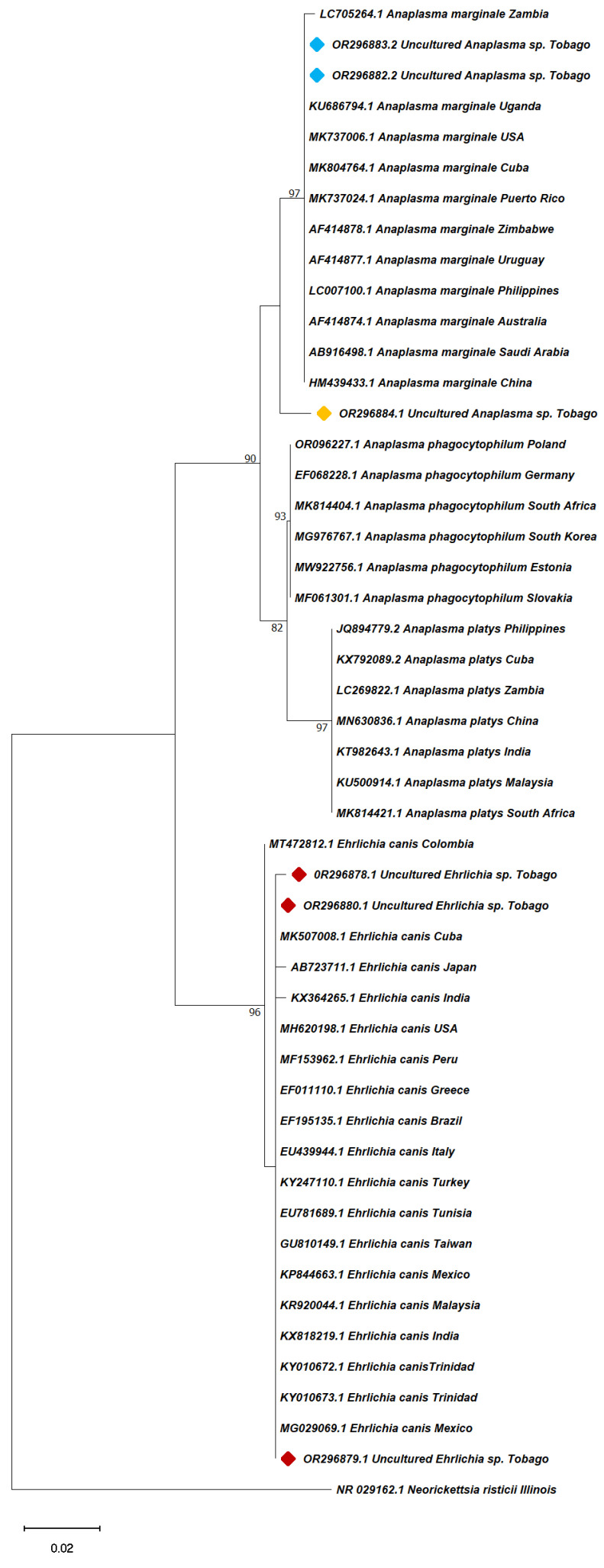
Phylogenetic tree of selected representatives of *Anaplasma* and *Ehrlichia* spp. inferred from 16S rRNA. The evolutionary history was inferred by using the maximum likelihood method and the Kimura 2-parameter as the best-fit model. The tree with the highest log likelihood (−861.12) is shown. The percentage of trees in which the associated taxa clustered together is shown next to the branches. Initial tree(s) for the heuristic search were obtained automatically by applying neighbour-joining and BioNJ algorithms to a matrix of pairwise distances estimated using the maximum composite likelihood (MCL) approach, and then selecting the topology with superior log likelihood value. The analysis contains *Ehrlichia* spp. The 16S rRNA sequences from dog blood (red diamonds; n = 3) and *Anaplasma* spp. sequences from dog blood (blue diamonds; n = 2) and an *A. ovale* tick (yellow diamond; n = 1), from Charlotteville, Tobago, together with nucleotide sequences from GenBank (no diamond; from canine blood), including Neorickettsia risticii as an outgroup. Sequence IDs are in the format accession number, pathogen and country of origin. Bootstrap values are represented as a per cent of internal branches (1000 replicates); values less than 70 are hidden. The tree is drawn to scale, with branch lengths measured in the number of substitutions per site. This analysis involved 49 nucleotide sequences. All positions containing gaps and missing data were eliminated (complete deletion option). There were a total of 343 positions in the final dataset. Evolutionary analyses were conducted in MEGA11.

A total of 49 nucleotide sequences were used to generate phylogenetic trees of *Ehrlichia* spp. (21) and *Anaplasma* spp. (27) with *Neorickettsia risticii* as an outgroup, while 21 sequences were used for *B. vogeli*. The 16S rRNA-based phylograms were computed to deduce the evolutionary relatedness of TBPs belonging to the *Ehrlichia*/*Anaplasma* genera (Figure 3). The Tobago *E. canis* sequences derived in this study clustered with published sequences from a broad range of geographic locations, including the Americas (including Trinidad), Africa, Asia and Europe, with an exception of one sequence (OR296881), which fell outside of the major *E. canis* clade (not included in tree).

**Figure 4:**
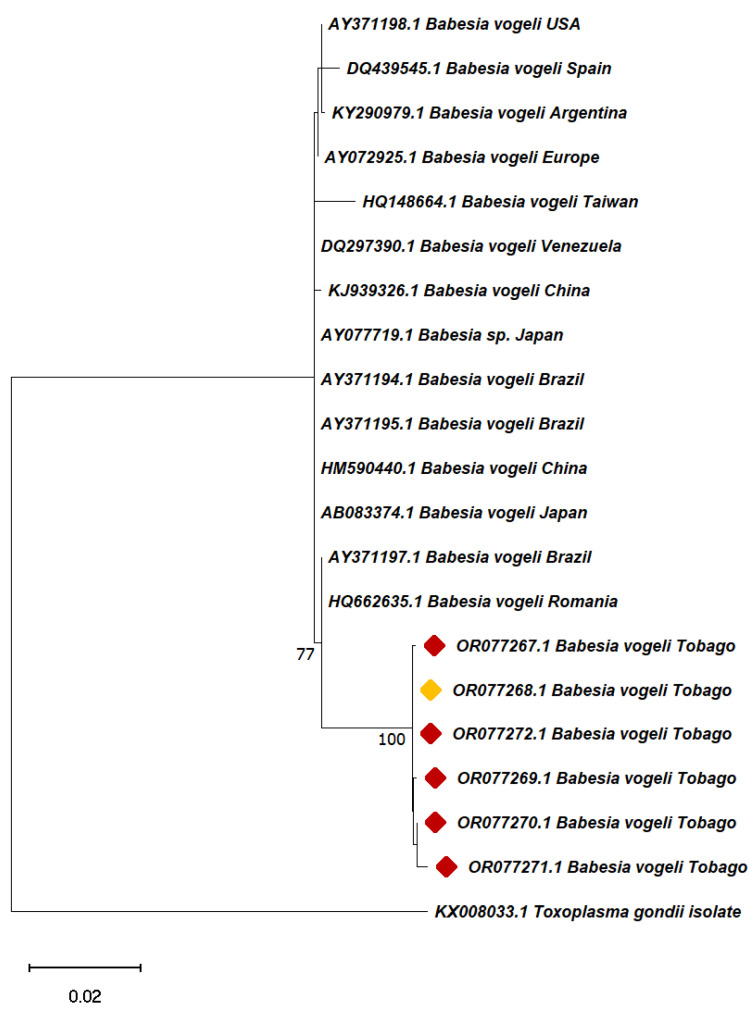
Phylogenetic tree of selected representatives of *Babesia vogeli* inferred from 18S rRNA. The evolutionary history was inferred by using the maximum likelihood method and Tamura–Nei as the best-fit model. The tree with the highest log likelihood (−3123.55) is shown. The percentage of trees in which the associated taxa clustered together is shown next to the branches. Initial tree(s) for the heuristic search were obtained automatically by applying neighbour-joining and BioNJ algorithms to a matrix of pairwise distances estimated using the Tamura–Nei model, and then selecting the topology with a superior log likelihood value. The analysis contains *Babesia vogeli* 18S rRNA sequences derived from *R. sanguineus* ticks (red diamonds; n = 5) and *A. ovale* ticks (yellow diamond; n = 1) from dogs in Charlotteville, Tobago, together with 15 sequences from GenBank (no diamonds; all from canine blood), including the *Toxoplasma gondii* sequence (GenBank KX008033.1) as an outgroup. Sequence IDs are in the format accession number, pathogen and country of origin. Bootstrap values are represented as per cent of internal branches (1000 replicates); values less than 70 are hidden. The tree is drawn to scale, with branch lengths measured in the number of substitutions per site. This analysis involved 21 nucleotide sequences. All positions containing gaps and missing data were eliminated (complete deletion option). There were a total of 1485 positions in the final dataset. Evolutionary analyses were conducted in MEGA11.

For the 18S rRNA-based phylogram, (Figure 4), *Babesia* spp. detected in this Tobago study clustered together in a strongly supported clade (bootstrap value = 100%) that was a sister clade to the one containing all other *B. vogeli* sequences.

## 4. Discussion

Haematophagous arthropods play a significant role in the transmission of diseases to animals and humans globally. In this study, we reported the detection of TBPs in a cluster of hunting dogs showing clinical signs suggestive of TBDs and in the ticks infesting them. To our knowledge, this is the first report of the simultaneous co-infection of dogs and ticks with protozoal and rickettsial TBPs on the island of Tobago. Additionally, this study demonstrates the first molecular detection of *Ehrlichia* spp., *Anaplasma* spp., *B. vogeli* and *H. canis* in dogs and/or ticks in Tobago, as previous molecular work on ticks from Tobago cattle and dogs focused on viral diversity [33].

The DNA of two rickettsial TBPs, *Ehrlichia* spp. (37.5%) and an *Anaplasma* spp. (25%) with 98–100% homology to *E. canis* and *A. marginale*, respectively, were detected in the blood of the Tobago dogs. From a previous report on the sister island of Trinidad, the molecular detection of *E. canis* in canine blood was 14.1% (49/348), while a subsequent serological survey reported almost 50% of the stray dog population being seropositive for this parasite [46,47]. A molecular study conducted on dogs in Grenada, reported *E. canis* (24.7%) and *A. platys* (19.2%) as the most prevalent TBPs in the blood of dogs [35]. *Ehrlichia canis* was also the most prevalent TBP detected in the blood of clinically suspected TBD cases (23.6%) and presumably healthy dogs (7.2%) from St. Kitts, West Indies, while *A. platys* was amplified in only apparently healthy dogs (3.6%) [48]. The relatively high frequency of *E. canis* in the Tobago cluster and other Caribbean islands corresponds with their tropical climate, which is conducive to the proliferation of the tick vector, *R. sanguineus* [49,50]. *Ehrlichia canis* was found in only one of the Tobago ticks (*A. ovale*) from a dog with *Anaplasma* spp. amplified in its blood. Considering that all stages of *A. ovale* can feed on canids, it could have acquired *E. canis* from a previous infected canine host.

The effects of CME is subclinical in some dogs while in others it is associated with leukopaenia, thrombocytopaenia (causes a bleeding diathesis) and anaemia [2,9]. It should be noted that, from our current study, two of the three dogs with *E. canis* showed similar clinical signs (anaemia, uveitis and ecchymotic haemorrhages). Paralysis and abortion was documented in the third dog, which have been reported in previous cases [51,52]. Dogs severely affected by CME may also present with marked pancytopaenia (leukopaenia, non-regenerative anaemia and thrombocytopaenia) due to bone marrow hypoplasia, as was the case with one of the dogs in the Tobago cluster [26]. Although there was molecular evidence of *E. canis* in the blood of these dogs, the organism was detected in only one of the stained blood smears. This is in agreement with a previous study which revealed that detection of the morula in monocytes is successful in only 4% of cases [53]. This finding justifies the need for molecular detection as a confirmatory diagnostic tool for CME.

*Anaplasma marginale*, the causative agent of bovine anaplasmosis, is a globally important TBP of cattle [54]. This pathogen is transmitted biologically by *Rhipicephalus* ticks and mechanically by biting flies and blood-contaminated fomites [55,56]. Fever exceeding 40 °C and anaemia are outstanding features of bovine anaplasmosis [57]. Although dogs are known to be hosts of *Anaplasma platys* and *Anaplasma phagocytophilum*, the DNA of *A. marginale* was detected in the blood of two dogs in this study. Interestingly, a previous study reported molecular evidence of *A. marginale* in the blood of two dogs from Hungary [58]. These dogs, similar to the dogs in our study were situated in a rural environment with free access to livestock, wildlife, their ticks and TBPs. This can possibly explain the presence of *A. marginale* DNA within the blood of the two dogs from Tobago. Further, the DNA of *A. marginale* was also amplified from one *A. ovale* tick from this study. The immature stages of *A. ovale* can be found on a range of small mammals and bird species, while adults complete their life cycle on larger mammals, including canids [12]. This tick may have possibly fed on an infected ruminant before attachment to its canid host. Further studies are, however, needed to elucidate the presence of *A. marginale* in the blood of these Tobago dogs.

Canine babesiosis is a globally significant TBP of protozoal origin. Molecular studies have detected *B. vogeli* and less frequently, B. gibsoni in dogs from various Caribbean islands [35,47,48,59]. The DNA of *B. vogeli* was detected in the blood of only one dog, co-infected with *E. canis*, in the Tobago cluster. Of note was the presence of rare intra-erythrocytic organisms morphologically similar to *Babesia* spp. in the blood of this same PCR-positive dog for *B. vogeli*. Clinically, this dog exhibited anaemia, anorexia, dermal ecchymoses and weight loss. These clinical signs were supported by a pancytopaenia on laboratory blood analysis. Although younger dogs are more likely to present with severe babesiosis, older dogs with co-infections, similar to this case, are also affected [4,60]. These findings highlight the importance of the concurrent use of molecular techniques in a clinical setting in detecting low parasitaemias for TBPs and the awareness of increased severity of clinical signs in dogs co-infected with TBPs [61].

*Hepatozoon canis* has been reported in dogs and ticks from the Caribbean and the Americas [34,35,62,63,64,65,66]. The DNA of *H. canis* was amplified in one of the *R. sanguineus* ticks but not in the host’s blood. As this tick was partially engorged, it can be surmised that it may have harboured the parasite before attachment to its current host. *Hepatozoon canis* is known to be transmitted via the ingestion of ticks, thus there is a possibility that this dog could have been infected by eating this infected tick. Other ixodids including *A. ovale* and *Rhipicephalus microplus* are vectors of *H. canis* and have been found infesting dogs and ruminants in Tobago [6,38,67,68].

## 5. Conclusions

The cases presented in this study appear to be a microcosm of the status of TBPs in Tobago, which is endemic in its neighbouring sister-island, Trinidad. Humans are also at risk of infection by these TBPs due to their close relationship with pets. As such, tick control is paramount in the prevention and control of TBDs in animals and humans. Veterinarians and physicians should be aware of the tick species present and their associated TBPs with inclusion of the latter as differentials for illnesses with similar clinical signs. Further, the importance of testing both the host and vector for the presence of TBPs using classical and molecular methods is warranted in completing the puzzle in epidemiological investigations related to TBDs.

## Figures and Tables

**Table 1:** Primer sequences used in this study.

Target Organism	Target Gene	Primer Name	Primer Sequence (5′-3′)	Product Size (bp)	Reference
*Ehrlichia*/ *Anaplasma*	16S rRNA	16 S8FE †	AGAGTTGGATCMTGGYTCAG	~500	[40]
		B-GA1B ‡	CGAGTTTGCCGGGACTTYTTC		[40]
*Babesia*/*Theileria*/ *Hepatozoon*	18S rRNA	RLB-F2 †	ACACAGGGAGGTAGTGACAAG	460–540	[39,41]
		RLB-R2 ‡	CTAAGAATTTCACCTCTGACAGT		[39,41]

† Forward primer; ‡ Reverse primer.

**Table 2:** Summary of clinical presentation and diagnostic investigation of affected dogs from Charlotteville, Tobago.

Case Number	Signalment	Clinical Presentation	Tick Species Detected (n = 20)	Haematology Results ^a^	Summary Haematology Report	Summary Biochemistry Report	Parasites on Blood Smear	Amplification of TBP DNA	
								Blood	Ticks
Dog 1	5 month old, intact female mixed breed	Anaemic Anorexic Listless Ecchymosis Icteric Paralysis (fore and hindlimbs) Weight loss	*Rhipicephalus sanguineus* (n = 2F, 1M)	WBC: 24.27 Segs: 13.24 RBC: 0.8 HCT: 0.043 Hgb: 16 PP: 45	Neutrophilia Lymphocytosis Poorly regenerative anaemia	Hypoproteinemia Hypoalbulinemia Hyponatremia, Hypochloremia Elevated BUN, ALT and CK	None detected	None	*Babesia vogeli*
Dog 2	7 month old, intact male mixed breed	Anorexic Listless Paralysis and oedema (fore and hindlimbs) Weight loss	*R. sanguineus* (n = 1M)	WBC: 30.38 Segs: 25.52 RBC: 5.46 HCT: 0.352 Hgb: 116	Mild, non-regenerative anaemia Neutrophilia Monocytosis	Hyperglobulinemia Elevated CK Hyperkalemia	None detected	None	None
Dog 3	10 month old, intact female hound	Anaemic Anorexic Listless Weight loss Thin blood	*R. sanguineus* (n = 3F, 3M)	WBC: 15.42 Segs: 13.26 PLT: 44	Neutrophilia Thrombocytopaenia	Hypoalbulinemia Hyperglobulinemia Hypocalcaemia	None detected	*Anaplasma* spp.	*B. vogeli* and *Hepatozoon canis*
Dog 4	2 year old, intact female hound	Anaemic Anorexic Listless Ecchymosis Uveitis	No ticks detected	WBC: 22.9 Segs: 6.41 RBC: 4.4 HCT: 0.283 Hgb: 98 PLT: 162	Non-regenerative anaemia Lymphocytosis, Monocytosis Cytotoxic T lymphocyte Clumped platelets	Hyperglobulinemia Hypoalbulinemia Elevated ALT and CK	None detected	*Ehrlichia* spp.	No ticks
Dog 5 §	3 year old, intact female hound	Anaemic Anorexic Ecchymosis Weight loss Thin blood	No ticks detected	WBC: 2.09 Segs: 1.8 RBC: 3.0 HCT: 0.184 Hgb: 60 PLT: 5	Pancytopenia (neutropenia, lymphopenia and thrombocytopaenia) Non-regenerative anaemia	Hyperglobulinemia Hypoalbulinemia Hyppocalcaemia Azotemia	*B.vogeli* and *Ehrlichia* spp.	*B. vogeli* and *Ehrlichia* spp.	No ticks
Dog 6 ‡§	4 year old, pregnant (6 weeks) hound	Aborted puppies Paralysis	*R. sanguineus* (n = 2M) *Amblyomma ovale* (n = 1F)	WBC: 9.4 Segs: 5.26 RBC: 5.92 HCT: 0.356 Hgb: 119 PLT: 100 PP: 87	Moderate platelet clumps Hyperproteinemia	Hyperproteinemia Hyperglobulinemia Hypoalbulinemia Elevated CK	None detected	*Ehrlichia* spp.	*Franciscella* endosymbiont
Dog 7 ‡§	6 year old, intact male hound	Anaemia Weight loss Paralysis	*R. sanguineus* (n = 4M) *A. ovale* (n = 2M)	WBC: 7.74 Segs: 5.73 RBC: 5.84 HCT: 0.34 Hgb: 115 PP: 80	Hyperproteinemia	Hyperproteinemia Hyperglobulinemia Hypoalbulinemia Elevated CK	None detected	None	*B. vogeli* and *Anaplasma* spp.
Dog 8 §	8 year old, intact female hound	Anaemia Listless Weight loss Paralysis	*A. ovale* (n = 1F)	WBC: 8.14 Segs: 5.05 RBC: 5.29 HCT: 0.343 Hgb: 113 PLT: 87	Non-regenerative anaemia Thrombocytopaenia	Hyperglobulinemia Hypoalbulinemia Hypocalcaemia Elevated CK	None detected	*Anaplasma* spp.	*B. vogeli* and *Ehrlichia* spp.

^a^ Complete blood count (CBC) reference intervals: White blood cell count (WBC) 6–17.1 × 10^9^/L; Neutrophils (Segs) 3.6–11.5 × 10^9^/L; Red blood cells (RBC) 5.5–8.3 × 10^12^/L; Haematocrit (HCT) 0.37–0.55 L/L, haemoglobin (Hgb) 120–180g/L; Platelets (PLT) 120–350 × 10^9^/L (46); Plasma proteins (PP) 55–76 g/L ‡ Same owner and kennel; § Dogs hunt together; LN lymph node, CK creatinine kinase, ALT alanine aminotransferase.

**Table 3:** Clinical outcome of the eight dogs sampled for tick-borne pathogens from Charlotteville, Tobago.

Case No.	Clinical Update/Outcome
Dog 1	Died on 13 November 2020. Carcass disposed of by owner.
Dog 2	Paralysis and other clinical signs resolved.
Dog 3	Resolution of clinical signs.
Dog 4	Bright, alert, responsive and eating well.
Dog 5	Much improvement after prednisone treatment and two cycles of doxycycline.
Dog 6	C-section done to remove six dead and decomposing pups. Treated for septic shock and doing better.
Dog 7	Treated for tick fever but still anorexic.
Dog 8	Died on 28 December 2020.

**Table 4:** Molecular detection of TBPs in canine hosts and tick vectors from a cluster of tick-fever suspected dogs from Charlotteville, Tobago.

Canine Host	TBPs in Host Blood	Tick ID	Tick spp.	TBPs in Ticks			
				16S rRNA		18S rRNA	
				*Ehrlichia* spp.	*Anaplasma* spp.	*Babesia vogeli*	*Hepatozoon canis*
Dog 1	n.d.	T1 T2 T3	*R.s* *R.s* *R.s*	- - -	- - -	- + -	- - -
Dog 2	n.d.	T4	*R.s*	-	-	-	-
Dog 3	*Anaplasma* spp.	T5 T6 T7 T8 T9 T10	*R.s* *R.s* *R.s* *R.s* *R.s* *R.s*	- - - - - -	- - - - - -	- + + - - -	- - - - - +
Dog 4	*Ehrlichia* spp.	No ticks	No ticks				
Dog 5	*Ehrlichia* spp. and *B. vogeli*	No ticks	No ticks				
Dog 6	*Ehrlichia* spp.	T11 T12 T13	*R.s* *R.s* *A.o*	- - -	- - -	- - -	- - -
Dog 7	n.d.	T14 T15 T16 T17 T18 T19	*R.s* *R.s* *R.s* *R.s* *A.o* *A.o*	- - - - - -	- - - - - +	- + + - - +	- - - - - -
Dog 8	*Anaplasma* spp.	T20	*A.o*	+	-	+	-

*R.s: Rhipicephalus sanguineus*; *A.o: *Amblyomma ovale**; n.d.: not detected.

**Table 5:** Comparison of DNA sequence similarities among pathogens detected in the Tobago dogs and ticks in this study and GenBank deposited sequences.

Pathogen Sequences from Ticks		Pathogen Sequences from Dog Blood	
Tobago TBP-Accession No. (Tick Id)	First GenBank Match TBP Accession No. (% identity)	Tobago TBP-Accession No. (Dog Id)	First GenBank Match TBP Accession No. (% identity)
*Babesia* spp.			
*Babesia vogeli*-OR077267.1 (T6)	*B. vogeli*-AY371197.1 (98)	*B. vogeli*-OR666420.1 (Dog 5)	*B. vogeli*-MN823219.1 (97)
*B. vogeli*-OR077268.1 (T19) ‡	*B. vogeli*-AY371197.1 (98)	-	-
*B. vogeli*-OR077269.1 (T2)	*B. vogeli*-AY371197.1 (98)	-	-
*B. vogeli*-OR077270.1 (T7)	*B. vogeli*-HM590440.1 (98)	-	-
*B. vogeli*-OR077271.1 (T15)	*B. vogeli*-HM590440.1 (98)	-	-
*B. vogeli*-OR077272.1 (T16)	*B. vogeli*-AY371197.1 (98)	-	-
*B. vogeli*-OR077273.1 (T20) ‡	*B. vogeli*-LC602472.1 (98)	-	-
*Hepatozoon* spp.			
*Hepatozoon canis*-OR077266.1 (T10)	*H. canis*-LC331053.1 (99)	-	-
		-	-
*Ehrlichia* spp.			
*Ehrlichia* spp.-OR29688.1 (T20) ‡	*E. canis*-KX364265.1 (98)	*Ehrlichia* spp.-OR296880.1 (Dog 4)	*E. canis*-AB287435.1 (100)
	-	*Ehrlichia* spp.-OR296878.1 (Dog 5)	*E. canis*-KY247110.1 (100)
	-	*Ehrlichia* spp.-OR296879.1 (Dog 6)	*E. canis*-AB287435.1 (100)
*Anaplasma* spp.			
*Anaplasma* spp.-OR296884.1 (T19) ‡	*A. marginale*-MK737024.1 (99)	-	-
*-*		-	-
*-*	-	*Anaplasma* spp.-OR296883.2 (Dog 3)	*A. marginale*-MK737024.1 (100)
*-*	-	*Anaplasma* spp.-OR296882.2 (Dog 8)	*A. marginale*-MK737024.1 (100)

‡ *Amblyomma ovale* tick; all others are *Rhipicehalus sanguineus*.

## Data Availability

Data not presented in this manuscript are available upon reasonable request.

## References

[1] Johnson E.M., Allen K.E., Panciera R.J., Ewing S.A., Little S.E. (2009). Experimental transmission of *Hepatozoon americanum* to New Zealand White rabbits (*Oryctolagus cuniculus*) and infectivity of cystozoites for a dog. Vet. Parasitol..

[2] Breitschwerdt E.B., Hegarty B.C., Hancock S.I. (1998). Sequential evaluation of dogs naturally infected with *Ehrlichia canis*, *Ehrlichia chaffeensis*, *Ehrlichia equi*, *Ehrlichia ewingii*, or *Bartonella vinsonii*. J. Clin. Microbiol..

[3] Birkenheuer A.J., Levy M.G., Savary K.C., Gager R.B., Breitschwerdt E.B. (1999). *Babesia gibsoni* infections in dogs from North Carolina. J. Am. Anim. Hosp. Assoc..

[4] Solano-Gallego L., Baneth G. (2011). Babesiosis in dogs and cats—Expanding parasitological and clinical spectra. Vet. Parasitol..

[5] Dantas-Torres F. (2010). Biology and ecology of the brown dog tick, *Rhipicephalus sanguineus*. Parasit. Vectors.

[6] Charles R., Basu A., Sanford B., King-Cenac A., Melville-Edwin S., Pow-Brown P., Sant C., Georges K. (2020). Survey of ticks of domestic dogs and cattle in three Caribbean islands. Transbound. Emerg. Dis..

[7] Dantas-Torres F. (2008). The brown dog tick, *Rhipicephalus sanguineus* (Latreille, 1806) (Acari: Ixodidae): From taxonomy to control. Vet. Parasitol..

[8] Dantas-Torres F. (2008). Canine vector-borne diseases in Brazil. Parasit. Vectors.

[9] Unver A., Perez M., Orellana N., Huang H., Rikihisa Y. (2001). Molecular and antigenic comparison of *Ehrlichia canis* isolates from dogs, ticks, and a human in Venezuela. J. Clin. Microbiol..

[10] Lineberry M.W., Grant A.N., Sundstrom K.D., Little S.E., Allen K.E. (2022). Diversity and geographic distribution of rickettsial agents identified in brown dog ticks from across the United States. Ticks Tick-Borne Dis..

[11] Gilot B., Laforge M., Pichot J., Raoult D. (1990). Relationships between the *Rhipicephalus sanguineus* complex ecology and Mediterranean spotted fever epidemiology in France. Eur. J. Epidemiol..

[12] Guglielmone A.A., Estrada-Pena A., Mangold A.J., Barros-Battesti D.M., Labruna M.B., Martins J.R., Venzal J.M., Arzua M., Keirans J.E. (2003). *Amblyomma aureolatum* (Pallas, 1772) and *Amblyomma ovale* Koch, 1844 (Acari: Ixodidae): Hosts, distribution and 16S rDNA sequences. Vet. Parasitol..

[13] Guglielmone A.A., Beati L., Barros-Battesti D.M., Labruna M.B., Nava S., Venzal J.M., Mangold A.J., Szabo M.P., Martins J.R., Gonzalez-Acuna D. (2006). Ticks (Ixodidae) on humans in South America. Exp. Appl. Acarol..

[14] Calderón V.Á., Fonseca V.H., Gamboa J.H. (2005). Catálogo de garrapatas suaves (Acari: Argasidae) y duras (Acari: Ixodidae) de Costa Rica. Brenesia.

[15] Bermudez C.S., Castro A., Esser H., Liefting Y., Garcia G., Miranda R.J. (2012). Ticks (Ixodida) on humans from central Panama, Panama (2010–2011). Exp. Appl. Acarol..

[16] Jaguezeski A.M., Lavina M.S., Orsolin V., da Silva A.S. (2018). *Amblyomma ovale* parasitizing a human. Comp. Clin. Pathol..

[17] da Paixao Seva A., Martins T.F., Munoz-Leal S., Rodrigues A.C., Pinter A., Luz H.R., Angerami R.N., Labruna M.B. (2019). A human case of spotted fever caused by *Rickettsia parkeri* strain Atlantic rainforest and its association to the tick *Amblyomma ovale*. Parasit. Vectors.

[18] Rubini A.S., Paduan K.S., Martins T.F., Labruna M.B., O’Dwyer L.H. (2009). Acquisition and transmission of *Hepatozoon canis* (Apicomplexa: Hepatozoidae) by the tick *Amblyomma ovale* (Acari: Ixodidae). Vet. Parasitol..

[19] Spolidorio M.G., Labruna M.B., Mantovani E., Brandao P.E., Richtzenhain L.J., Yoshinari N.H. (2010). Novel spotted fever group rickettsiosis, Brazil. Emerg. Infect. Dis..

[20] Silva N., Eremeeva M.E., Rozental T., Ribeiro G.S., Paddock C.D., Ramos E.A., Favacho A.R., Reis M.G., Dasch G.A., de Lemos E.R. (2011). Eschar-associated spotted fever rickettsiosis, Bahia, Brazil. Emerg. Infect. Dis..

[21] Krawczak F.S., Muñoz-Leal S., Guztzazky A.C., Oliveira S.V., Santos F.C., Angerami R.N., Moraes-Filho J., de Souza J.C., Labruna M.B. (2016). Case report: *Rickettsia* sp. strain atlantic rainforest infection in a patient from a spotted fever-endemic area in southern Brazil. Am. J. Trop. Med. Hyg..

[22] Gondard M., Cabezas-Cruz A., Charles R.A., Vayssier-Taussat M., Albina E., Moutailler S. (2017). Ticks and Tick-Borne Pathogens of the Caribbean: Current Understanding and Future Directions for More Comprehensive Surveillance. Front. Cell Infect. Microbiol..

[23] Ewing S.A., Panciera R.J. (2003). American canine hepatozoonosis. Clin. Microbiol. Rev..

[24] Harvey J.W., Simpson C.F., Gaskin J.M. (1978). Cyclic thrombocytopenia induced by a Rickettsia-like agent in dogs. J. Infect. Dis..

[25] Sainz A., Roura X., Miro G., Estrada-Pena A., Kohn B., Harrus S., Solano-Gallego L. (2015). Guideline for veterinary practitioners on canine ehrlichiosis and anaplasmosis in Europe. Parasit. Vectors.

[26] Harrus S., Bark H., Waner T. (1997). Canine monocytic ehrlichiosis: An update. Compend. Contin. Educ. Pract. Vet..

[27] Harrus S., Waner T. (2011). Diagnosis of canine monocytotropic ehrlichiosis (*Ehrlichia canis*): An overview. Vet. J..

[28] Dumler J.S., Barbet A.F., Bekker C., Dasch G.A., Palmer G.H., Ray S.C., Rikihisa Y., Rurangirwa F.R. (2001). Reorganization of genera in the families Rickettsiaceae and Anaplasmataceae in the order Rickettsiales: Unification of some species of *Ehrlichia* with Anaplasma, Cowdria with *Ehrlichia* and *Ehrlichia* with Neorickettsia, descriptions of six new species combinations and designation of *Ehrlichia equi* and ‘HGE agent’ as subjective synonyms of *Ehrlichia phagocytophila*. Int. J. Syst. Evol. Microbiol..

[29] Snellgrove A.N., Krapiunaya I., Ford S.L., Stanley H.M., Wickson A.G., Hartzer K.L., Levin M.L. (2020). Vector competence of *Rhipicephalus sanguineus* sensu stricto for *Anaplasma platys*. Ticks Tick-Borne Dis..

[30] Lanza-Perea M., Zieger U., Qurollo B.A., Hegarty B.C., Pultorak E.L., Kumthekar S., Bruhl-Day R., Breitschwerdt E.B. (2014). Intraoperative bleeding in dogs from Grenada seroreactive to *Anaplasma platys* and *Ehrlichia canis*. J. Vet. Intern. Med..

[31] Alhassan A., Hove P., Sharma B., Matthew-Belmar V., Karasek I., Lanza-Perea M., Werners A.H., Wilkerson M.J., Ganta R.R. (2021). Molecular detection and characterization of *Anaplasma platys* and *Ehrlichia canis* in dogs from the Caribbean. Ticks Tick-Borne Dis..

[32] Almazan C., Gonzalez-Alvarez V.H., Fernandez de Mera I.G., Cabezas-Cruz A., Rodriguez-Martinez R., de la Fuente J. (2016). Molecular identification and characterization of *Anaplasma platys* and *Ehrlichia canis* in dogs in Mexico. Ticks Tick-Borne Dis..

[33] Sameroff S., Tokarz R., Charles R.A., Jain K., Oleynik A., Che X., Georges K., Carrington C.V., Lipkin W.I., Oura C. (2019). Viral diversity of tick species parasitizing cattle and dogs in Trinidad and Tobago. Sci. Rep..

[34] Sant C., Georges K.C., Pow-Brown P. (2017). Novel incidental finding of *Hepatozoon canis* infection in two dogs of the same household in Trinidad, West Indies. Vet. Parasitol. Reg. Stud. Rep..

[35] Yabsley M.J., McKibben J., Macpherson C.N., Cattan P.F., Cherry N.A., Hegarty B.C., Breitschwerdt E.B., O’Connor T., Chandrashekar R., Paterson T. (2008). Prevalence of *Ehrlichia canis*, *Anaplasma platys*, *Babesia canis vogeli*, *Hepatozoon canis*, *Bartonella vinsonii berkhoffii*, and *Rickettsia* spp. in dogs from Grenada. Vet. Parasitol..

[36] Sharma B., Ganta R.R., Stone D., Alhassan A., Lanza-Perea M., Matthew Belmar V., Karasek I., Cooksey E., Butler C.M., Gibson K. (2021). Development of a multiplex PCR and magnetic DNA capture assay for detecting six species pathogens of the genera *Anaplasma* and *Ehrlichia* in canine, bovine, caprine and ovine blood samples from Grenada, West Indies. Pathogens.

[37] Alvarez D.O., Corona-Gonzalez B., Rodriguez-Mallon A., Rodriguez Gonzalez I., Alfonso P., Noda Ramos A.A., Diaz-Sanchez A.A., Gonzalez Navarrete M., Rodriguez Fernandez R., Mendez Mellor L. (2020). Ticks and Tick-Borne Diseases in Cuba, Half a Century of Scientific Research. Pathogens.

[38] Basu A.K., Charles R. (2017). Ticks of Trinidad and Tobago—An Overview.

[39] Gubbels J.M., de Vos A.P., van der Weide M., Viseras J., Schouls L.M., de Vries E., Jongejan F. (1999). Simultaneous detection of bovine *Theileria* and *Babesia* species by reverse line blot hybridization. J. Clin. Microbiol..

[40] Bekker C.P., de Vos S., Taoufik A., Sparagano O.A., Jongejan F. (2002). Simultaneous detection of *Anaplasma* and *Ehrlichia* species in ruminants and detection of *Ehrlichia ruminantium* in *Amblyomma variegatum* ticks by reverse line blot hybridization. Vet. Microbiol..

[41] Schouls L.M., Van De Pol I., Rijpkema S.G., Schot C.S. (1999). Detection and identification of *Ehrlichia*, *Borrelia burgdorferi* sensu lato, and *Bartonella* species in Dutch *Ixodes ricinus* ticks. J. Clin. Microbiol..

[42] Weiss D.J., Wardrop K.J. (2011). Schalm’s Veterinary Hematology.

[43] Tamura K., Stecher G., Kumar S. (2021). MEGA11: Molecular Evolutionary Genetics Analysis Version 11. Mol. Biol. Evol..

[44] Tamura K., Nei M. (1993). Estimation of the number of nucleotide substitutions in the control region of mitochondrial DNA in humans and chimpanzees. Mol. Biol. Evol..

[45] Comazzi S., Pieralisi C., Bertazzolo W. (2004). Haematological and biochemical abnormalities in canine blood: Frequency and associations in 1022 samples. J. Small Anim. Pract..

[46] Asgarali Z., Pargass I., Adam J., Mutani A., Ezeokoli C. (2012). Haematological parameters in stray dogs seropositive and seronegative to *Ehrlichia canis* in North Trinidad. Ticks Tick-Borne Dis..

[47] Georges K., Ezeokoli C.D., Newaj-Fyzul A., Campbell M., Mootoo N., Mutani A., Sparagano O.A. (2008). The application of PCR and reverse line blot hybridization to detect arthropod-borne hemopathogens of dogs and cats in Trinidad. Ann. N. Y. Acad. Sci..

[48] Loftis A.D., Kelly P.J., Freeman M.D., Fitzharris S., Beeler-Marfisi J., Wang C. (2013). Tick-borne pathogens and disease in dogs on St. Kitts, West Indies. Vet. Parasitol..

[49] Keefe T., Holland C., Salyer P., Ristic M. (1982). Distribution of *Ehrlichia canis* among military working dogs in the world and selected civilian dogs in the United States. J. Am. Vet. Med. Assoc..

[50] Ewing S. (1972). Geographic distribution and tick transmission of *Ehrlichia canis*. J. Med. Entomol..

[51] Codner E., Farris-Smith L. (1986). Characterization of the subclinical phase of ehrlichiosis in dogs. J. Am. Vet. Med. Assoc..

[52] Cowell R., Tyler R., Clinkenbeard K., Meinkoth J. (1988). Ehrlichiosis and polyarthritis in three dogs. J. Am. Vet. Med. Assoc..

[53] Woody B.J., Hoskins J.D. (1991). Ehrlichial diseases of dogs. Vet. Clin. N. Am. Small Anim. Pract..

[54] Jongejan F., Uilenberg G. (2004). The global importance of ticks. Parasitology.

[55] Dikmans G. (1950). The transmission of anaplasmosis. Am. J. Vet. Res..

[56] Kocan K.M., De La Fuente J., Blouin E., Garcia-Garcia J. (2004). Anaplasma marginale (Rickettsiales: Anaplasmataceae): Recent advances in defining host–pathogen adaptations of a tick-borne rickettsia. Parasitology.

[57] Kocan K.M., de la Fuente J., Blouin E.F., Coetzee J.F., Ewing S. (2010). The natural history of *Anaplasma* marginale. Vet. Parasitol..

[58] Hornok S., Horvath G., Takacs N., Farkas R., Szoke K., Kontschan J. (2018). Molecular evidence of a badger-associated *Ehrlichia* sp., a Candidatus Neoehrlichia lotoris-like genotype and *Anaplasma marginale* in dogs. Ticks Tick-Borne Dis..

[59] Kelly P.J., Xu C., Lucas H., Loftis A., Abete J., Zeoli F., Stevens A., Jaegersen K., Ackerson K., Gessner A. (2013). Ehrlichiosis, babesiosis, anaplasmosis and hepatozoonosis in dogs from St. Kitts, West Indies. PLoS ONE.

[60] Solano-Gallego L., Trotta M., Carli E., Carcy B., Caldin M., Furlanello T. (2008). *Babesia canis canis* and *Babesia canis vogeli* clinicopathological findings and DNA detection by means of PCR-RFLP in blood from Italian dogs suspected of tick-borne disease. Vet. Parasitol..

[61] Shaw S.E., Day M.J., Birtles R.J., Breitschwerdt E.B. (2001). Tick-borne infectious diseases of dogs. Trends Parasitol..

[62] Diaz-Sanchez A.A., Hofmann-Lehmann R., Meli M.L., Roblejo-Arias L., Fonseca-Rodriguez O., Castillo A.P., Canizares E.V., Rivero E.L., Chilton N.B., Corona-Gonzalez B. (2021). Molecular detection and characterization of *Hepatozoon canis* in stray dogs from Cuba. Parasitol. Int..

[63] Thomas R., Santodomingo A., Castro L. (2020). Molecular detection of *Babesia canis vogeli* and *Hepatozoon canis* in dogs in the department of Magdalena (Colombia). Rev. Fac. Med. Vet. Zootec..

[64] Wei L., Kelly P., Ackerson K., El-Mahallawy H.S., Kaltenboeck B., Wang C. (2015). Molecular detection of *Dirofilaria immitis*, *Hepatozoon canis*, *Babesia* spp., *Anaplasma platys*, and *Ehrlichia canis* in dogs on Costa Rica. Acta Parasitol..

[65] Vilar T., Volino W., Nalim M., Barros N., Stelling W., Serra-Freire N., Almosny N. (2005). Registro de Infeccao no Rio de Janeiro Brasil por *Hepatozoon* sp. e *Ehrlichia* sp. em cao (*Canis familiaris*) Proveniente de Aruba, Caribe. South American Conference of Veterinary Medicine Proceedings.

[66] Starkey L.A., Newton K., Brunker J., Crowdis K., Edourad EJ P., Meneus P., Little S.E. (2016). Prevalence of vector-borne pathogens in dogs from Haiti. Vet. Parasitol..

[67] de Miranda R.L., de Castro J.R., Olegario M.M., Beletti M.E., Mundim A.V., O’Dwyer L.H., Eyal O., Talmi-Frank D., Cury M.C., Baneth G. (2011). Oocysts of *Hepatozoon canis* in *Rhipicephalus* (*Boophilus*) *microplus* collected from a naturally infected dog. Vet. Parasitol..

[68] de Castro Demoner L., Rubini A.S., dos Santos Paduan K., Metzger B., de Paula Antunes J.M.A., Martins T.F., Mathias M.I.C., O’Dwyer L.H. (2013). Investigation of tick vectors of *Hepatozoon canis* in Brazil. Ticks Tick-Borne Dis..

